# Epigenetic Aspects of a New Probiotic Concept—A Pilot Study

**DOI:** 10.3390/life13091912

**Published:** 2023-09-14

**Authors:** Nina Okuka, Verena Schuh, Ulrike Krammer, Snezana Polovina, Mirjana Sumarac-Dumanovic, Neda Milinkovic, Ksenija Velickovic, Brizita Djordjevic, Alexander Haslberger, Nevena Dj. Ivanovic

**Affiliations:** 1 Department of Bromatology, Faculty of Medicine, University of Banja Luka, 78000 Banja Luka, Bosnia and Herzegovina; nina.vukicevic@med.unibl.org; 2 HealthBioCare GmbH, 1090 Vienna, Austria; 3 Clinic for Endocrinology, Diabetes and Diseases of Metabolism, Clinical Center of Serbia, 11000 Belgrade, Serbia; 4 School of Medicine, University of Belgrade, Clinic for Endocrinology, Diabetes and Diseases of Metabolism, 11000 Belgrade, Serbia; 5 Department of Medical Biochemistry, Faculty of Pharmacy, University of Belgrade, 11000 Belgrade, Serbia; 6 Department of Cell and Tissue Biology, Faculty of Biology, University of Belgrade; 11000 Belgrade, Serbia; 7 Department of Bromatology, Faculty of Pharmacy, University of Belgrade, 11000 Belgrade, Serbia; 8 Department of Nutritional Science, University of Vienna, 1090 Vienna, Austria

**Keywords:** obesity, probiotics, epigenetic markers, mRNA, miRNAs, SIRT1, *Lactobacillus plantarum* 299v, *Saccharomyces boulardii* var. *cerevisiae*, policosanols

## Abstract

Several studies report the important role of an altered gut microbiota in the development of obesity, highlighting the potential use of probiotics in the treatment of obesity. The aim of this study is to investigate the effect of a novel probiotic approach on the expression of specific miRNAs and mRNAs associated with obesity in combination with the hypocholesterolemic octacosanol. Twenty overweight/obese women participated in a randomized, placebo-controlled, double-blind study and were randomly divided into two groups: the intervention group (daily one capsule containing *Lactobacillus plantarum* 299v (DSM9843), *Saccharomyces cerevisiae* var. *boulardii*, and 40 mg octacosanol; N = 12) and the placebo group (N = 8). Changes in lipid parameters and expression of miRNAs and mRNAs were assessed before (T0) and after the 12-week intervention (T1). After the intervention, the expression of miR-155-5p (9.38 ± 0.85 vs. 8.38 ± 1.06, *p* = 0.05) and miR-24-3p (3.42 ± 0.38 vs. 2.71 ± 0.97, *p* = 0.031) showed significant decreases in the intervention group when compared to the control group. At T1, the expression of miR-155-5p (8.69 ± 1.31 vs. 9.3 ± 0.85, *p* = 0.04), miR-125b-5p (5.41 ± 1.18 vs. 5.99 ± 1.36, *p* = 0.049), and *TNF-α* (10.24 ± 1.66 vs. 11.36 ± 1.12, *p* = 0.009) were significantly decreased in the intervention group. No changes in lipids and anthropometric parameters were observed. The novel probiotic approach had a positive effect on regulating the expression of certain miRNAs and mRNAs important for regulating inflammation and adipogenesis, which are essential for obesity onset and control.

## 1. Introduction

The incidence of obesity has increased dramatically worldwide over the past four decades, making obesity a major contributor to the global burden of chronic disease and disability in all age groups [1]. Obesity is the result of altered energy balance, i.e., the way the body regulates energy intake, consumption, and storage. However, other factors, such as genetic predisposition and environmental factors, also contribute significantly to the development of obesity [2]. In the last decade, the gut microbiota has been identified as one of the influential environmental factors that play a critical role in the development and progression of obesity and obesity-related diseases, particularly in terms of changes in microbiota composition and metabolites during the progression of obesity [3]. Although lifestyle interventions remain the primary strategy for treating obesity and its associated diseases, novel therapies that target one or more of the underlying aetiological factors are desirable. Evidence from the past decade supports the idea that a new approach to treating obesity could involve influencing the composition of the microbiota or correcting dysbiosis [4]. Research on probiotics has made progress in identifying the precise molecular mechanisms underlying the health-promoting properties of probiotics, which are still poorly understood [5,6]. In particular, the results of several studies suggest that epigenetic influences may be mediators of the interaction between the host and the microbiome or probiotics [5,6]. For example, microRNAs (miRNAs), small and non-coding RNAs, have been identified as important molecular mediators of the host–microbiome interaction. In addition, there are other studies in the literature suggesting that possible mechanisms of action of probiotics are mediated via miRNAs [6,7]. Furthermore, it has been suggested that epigenetic DNA methylation may also be one of the molecular mechanisms by which probiotics exert their effects on the host [8]. Some studies also reported that the beneficial effects of probiotics are mediated by the activation of sirtuins, particularly SIRT1. SIRT1 is a highly conserved NAD^+^-dependent protein deacetylases/ADP ribosyltransferases whose activation has been shown to improve glucose tolerance, regulate lipid and cholesterol metabolism, and play a critical role in suppressing inflammation [9,10], a state that is associated with obesity.

In addition to probiotics, there are a large number of biologically active compounds (nutraceuticals) that may be useful in the prevention and treatment of risk factors associated with obesity, such as dyslipidaemia, increased platelet aggregation, inflammation, etc. The use of policosanol, a natural mixture of aliphatic primary alcohols isolated from purified sugar cane, can be considered one of these substances.

The individual components of the new probiotic concept used in our study exert different actions that, together, could have a synergistic effect in treating obesity and the associated metabolic profile. The probiotic strain *L. plantarum* 299v is a well-characterised strain that has demonstrated the nonpathogenic, anti-inflammatory, and hypocholesterolaemic effects in humans [11,12]. *S. boulardii* is also a well-characterised yeast strain classified as a probiotic. The beneficial effects of using *S. boulardii* are associated with specific mechanisms of action, such as antimicrobial activity, a trophic effect on the intestinal mucosa, stimulation of the immune response, and increased production of butyrate [13]. However, potential positive effects of this yeast strain in the treatment of obesity have only been discussed in several papers. The results of an animal study showed that *S. boulardii* can influence the reduction of body mass, body fat, hepatic steatosis, and inflammation [13]. Policosanols have been reported to be effective in lowering LDL cholesterol and triglycerides (TG) at a dose of about 20 mg daily. In addition to lowering low-density lipoprotein (LDL) cholesterol, policosanols may also exert a beneficial effect on high-density lipoprotein (HDL) cholesterol [14,15]. Furthermore, literature data indicate that policosanol may contribute to the reduction of platelet aggregation, endothelial damage, and oxidised LDL (oxLDL) formation [16,17]. According to the aforementioned literature data, the combination of *L. plantarum* 299v, *S. boulardii*, and policosanol may have a synergistic effect in the treatment of obesity within the context of a healthy lifestyle. 

Considering the increasing demand in the market and health care system for dietary supplements and nutraceuticals that provide effective and safe support for medical nutrition therapy in obesity, the main objective of this study is to investigate the effects of a new probiotic product on the expression of certain miRNAs and mRNAs crucial for regulating inflammation and adipogenesis, essential factors in obesity onset and control.

## 2. Materials and Methods

### 2.1. Subjects 

Twenty overweight (BMI = 25–30 kg/m^2^) or obese (BMI ≥ 30 kg/m^2^) women of childbearing age (29–50 years old) were recruited for this study. Recruitment took place at the Centre for Obesity, Clinic for Endocrinology, Diabetes and Metabolic Diseases, Clinical Centre of Serbia, Belgrade. Exclusion criteria were patients younger than 18 years, women in menopause, chronic kidney, thyroid, and gastrointestinal diseases (Crohn’s disease, ulcerative colitis), patients who had taken probiotic, prebiotic, or antibiotic therapy in the last month, hypersensitivity/intolerance to any of the ingredients of the product, pregnancy or breastfeeding, and that have not given birth to a child in the last year. According to the data from the questionnaire, the patients did not use drugs or undergo surgical interventions for weight loss.

### 2.2. Study Design 

The study was conducted as a randomised, double-blind, placebo-controlled trial. Assignment to the intervention or placebo group was concealed from the researchers, and the probiotic and placebo capsules both had an identical appearance. Therefore, neither the subjects nor the investigators knew which treatment they were receiving in this study. A total of 25 subjects met the inclusion criteria; 5 of them were excluded because they were not willing to participate in the study or because they had taken antibiotics during the study.

The participants were divided into two groups. The first group of participants (probiotic/intervention group) received an oral capsule containing 7 × 10^10^ CFU *Lactobacillus plantarum* 299v (DSM9843), 5 × 10^9^ CFU *Saccharomyces cerevisiae* var. *boulardii*, and 40 mg octacosanol once daily for 3 months, and another group of patients (placebo/control group) received an oral capsule containing placebo (maltodextrin) once daily for 3 months. Patients were given a 12-week supply of probiotic or placebo capsules at the start of the study and were asked to take one capsule daily after breakfast. Adherence to taking the capsules was monitored once a month through telephone interviews, and patients were asked to bring back any capsules they had not taken at the end of the study. In addition, participants were given advice on healthy eating and lifestyle habits to follow during the intervention phase.

All participants signed a written informed consent form. This study was approved by the Ethics Committee of the Clinical Centre of Serbia (number 31/28, dated 21 February 2019). Our study has been registered with the Australian New Zealand Clinical Trials Registry (ANZCTR). The registration number is ACTRN12622000696796.

### 2.3. Data Collection

Anthropometric measurements, blood samples, and information on food intake (using the FFQ) were collected at two intervals: at baseline (T0) and at the end of week 12 (T1). The FFQ included questions about the frequency of consuming foods rich in dietary fibres, probiotics, and prebiotics (i.e., fruits, vegetables, whole grains, dairy products), as well as questions about lifestyle factors (i.e., frequency of physical activity, smoking habits, alcohol consumption, use of dietary supplements). Anthropometric measurements were performed by trained personnel using the Tanita Body Composition Analyser BC-420MA. Blood samples were taken in the early morning (between 7:00 a.m. and 9:00 a.m.) after a 12 h overnight fasting period and analysed at the beginning of the study and after the intervention.

### 2.4. Biochemical Parameters

The biochemical parameters were determined in the blood samples. Standard operating procedures were followed for collection and classification of blood samples [18]. A Becton Dickinson closed venipuncture system (BD) was used to collect blood samples (Vacutainer, 22 standard wire gauge (SWG), and reusable adapters). Serum samples were collected using serum separator vacutainers with clot activator (BD Vacutainer^®^ SST™ Tubes).

All biochemical parameters were determined using the Olympus AU400 clinical chemistry analyser (Beckman Coulter Diagnostic, Inc., Brea, CA, USA). Concentrations of total cholesterol (TC), HDL, LDL, and triglycerides (TG) were measured in serum using the spectrophotometry method. Precision and accuracy for all analysed parameters were verified using commercial certified reference material. 

### 2.5. Preparation of Dried Blood Spots

Participants’ capillary blood was collected from the fingertip of the non-dominant hand onto Whatman^®^ Protein Saver cards (Sigma-Aldrich, Vienna, Austria) using the Extra 18G safety lancet (Sarstedt, Germany). These dried blood spots (DBS) were stored at −20 °C.

### 2.6. DNA/RNA Extraction

Four 4 mm diameter punches were cut out of each DBS and placed in a protease solution. Then samples were incubated overnight in a ThermoMixer. DNA and RNA were extracted using the KingFisher^TM^ Duo Prime Purification System and MagMAXTM FFPE DNA/RNA Ultra Kit according to the manufacturer’s protocol (Applied Biosystems™ by Thermo Fisher Scientific, Waltham, MA, USA).

### 2.7. cDNA Synthesis miRNA and mRNA

To prepare templates for the detection and quantification of miRNAs, the extracted RNA must be transcribed into complementary DNA (cDNA). The cDNA synthesis with the steps poly(A)-tailing reaction, adaptor ligation reaction, reverse transcription reaction, and miR-Amp reaction was performed using the SimpliAmp^TM^ Thermal Cycler (Applied Biosystems™ by Thermo Fisher Scientific) and the TaqManTM Advanced miRNA cDNA Synthesis Kit according to the manufacturer’s protocol (Applied Biosystems™ by Thermo Fisher Scientific). The cDNA templates were diluted 1:10 and then stored at −20 °C. The cDNA of the mRNA was prepared using the LunaScript RT SuperMix Kit according to the manufacturer’s protocol (Biolabs, Heidelberg, Germany).

### 2.8. mRNA and miRNA Expression

Changes in miRNA and mRNA expression for genes associated with obesity and inflammation (*SIRT1*, *PDK4*, *TNFα*) were determined using commercially available primers (Thermofisher, Waltham, MA, USA). All target mRNAs were normalised to β-actin and all miRNAs were normalised to miR-93-5p as an endogenous control. For the determination of miRNAs and mRNAs, a PCR reaction mix was prepared for each miRNA containing TaqMan^TM^ Fast Advanced Master Mix (Applied Biosystems™ by Thermo Fisher Scientific), TaqMan^TM^ Advanced miRNA or mRNA Assay (Applied Biosystems™ by Thermo Fisher Scientific) and nuclease-free water. The real-time polymerase chain reaction (qPCR) was performed using the QuantStudio^TM^ 3 Real-Time PCR System under standard settings (Applied Biosystems™ by Thermo Fisher Scientific). The generated data were analysed using the Applied Biosystems™ Real-Time PCR Analysis Modules (by Thermo Fisher Scientific). The ΔCt method was used for the following expression calculation. The mean Ct value of each sample was normalised to the mean Ct value of the housekeeping gene miR-93-5p to obtain ΔCt. 

### 2.9. Statistical Analysis

All data are expressed as mean ± standard deviation (SD). Data analysis was performed using the statistical software IBM SPSS v23.0 (IBM Corp., Armonk, NY, USA). MANOVA and ANOVA repeated measures 2 × 2 protocol was used for differences between groups and different time points. Partial Eta2 was used to determine the effect of supplementation on differences within and between groups. The difference in mean values of variables related to supplementation in each group was expressed as a delta value in %. The delta value is calculated according to the formula: ((T1/T0) − 1) − 100. Correlations between dietary style, lipids, and molecular parameters were tested using Spearman’s correlation. A *p*-value ≤ 0.05 was considered statistically significant for all tests. 

## 3. Results

### 3.1. Dietary Habits Analysis

The analysis of the food frequency questionnaire revealed no significant differences in the consumption of processed foods, fruits and vegetables, meat, fish, cereal products, and sweets between the placebo and intervention groups. Only the consumption of dairy products showed a difference. Physical activity levels varied between the groups, with women in the placebo group reporting more physical activity, which included walking, gardening, shopping, and vacuuming. Women in both groups reported that they rarely engaged in more than 30 min of vigorous physical activity per day (Table 1).

### 3.2. Anthropometric Changes

At baseline, subjects in both intervention and control groups were equal in terms of anthropometric characteristics, including mean weight, mean BMI, and mean body fat mass. No significant changes within or between the groups were observed after the intervention (Table 2). The last columns of Table 2 and Table 3 show the percentage change within group between T0 and T1. The results are presented as delta (Δ) values, expressed as a percentage of change in some parameters. There were no statistically significant differences between the changes in anthropometric measurements (weight, BMI, body fat mass).

### 3.3. Analysis of Lipid Parameter Changes

The subjects in both groups had a uniform lipid profile at the beginning of the study. However, probiotic supplementation did not lead to significant changes in lipid parameters within or between the groups (Table 3).

### 3.4. Molecular Changes following 12 Weeks of Intervention

Expression of selected miRNAs and mRNAs were determined in capillary blood before and after the intervention. Twelve weeks of probiotic supplementation significantly decreased the expression of miR-155-5p (*p* = 0.040), miR-125b-5p (*p* = 0.049), and *TNF-α* mRNA (*p* = 0.009). MiR-26b-5p was down-regulated, but only with a *p*-value of 0.067. In addition, the expression of miR-126-3p, miR-34a-5p, miR-24-3p, *SIRT1*, and *PDK4* was not statistically altered by probiotic supplementation (Table 4). Compared to the control group, the expression of miR-155-5p and miR-24-3p were significantly decreased in the intervention group after the intervention (Table 4). The expression of the other miRNAs and mRNAs tested were not statistically different between the groups.

### 3.5. Correlations between Dietary Habits and Anthropometric Data with Selected Epigenetic Markers

**Figure 1:**
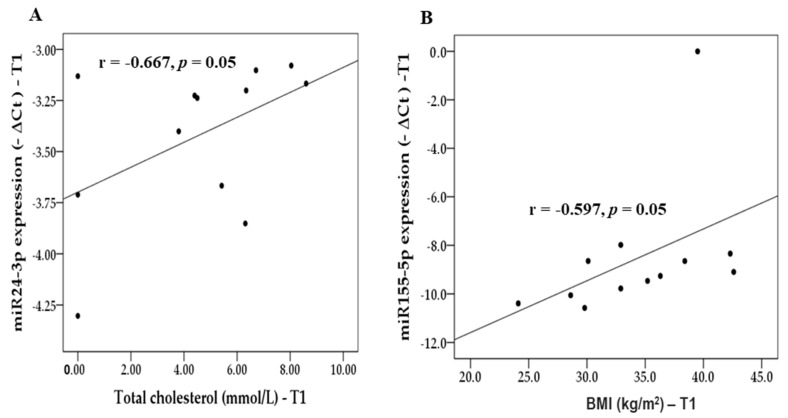
Correlations after the intervention. (**A**) Correlation between total cholesterol (TC, mmol/L) and miR24-3p expression (−ΔCt). (**B**) Correlation between Body Mass Index (BMI, kg/m^2^) and miR155-5p expression (−ΔCt).

After the intervention, positive correlation was found between the miR-24-3p expression and TC (r = −0.667, *p* = 0.05, Figure 1A), and BMI positively correlated with miR-155-5p expression (r = −0.597, *p* = 0.05; Figure 1B). For example, patients whose BMIs were lower had lower miR-155-5p expression, and patients whose TC levels were lower had lower miR-24-3p expression.

## 4. Discussion

There is a growing body of evidence supporting the efficacy of probiotics in the prevention and alleviation of obesity and related metabolic diseases [19]. Considering recent findings that one of the possible mechanisms of probiotic action is mediated by epigenetic influences, the aim of this study was to investigate the effect of a new probiotic concept on selected epigenetic markers specific to inflammatory and metabolic diseases.

Considering that obesity and overweight are strongly associated with dyslipidaemia, this study determined the levels of total cholesterol, LDL, HDL, and triglycerides in the blood. Although several studies have reported the lipid-regulating effect of different probiotic strains in obese patients, the data are not consistent [20]. In our study, no significant changes in lipid parameters were observed within or between groups at baseline and after the intervention (Table 3). The possible explanation could be a lower dose used here, since Qui et al. showed that Lactobacillus supplementation at a dose of ≥10^10^ CFU/day for at least 12 weeks was able to lower LDL and total cholesterol levels but not TG. 

It is well known that that obesity is considered to be a state of low-grade chronic inflammation and that macrophages play a crucial role in the synthesis of proinflammatory cytokines and certain proinflammatory miRNAs. Increased serum and plasma levels of miR-155 have been found in many diseases, such as type 2 diabetes (T2D), metabolic syndrome, obesity, atherosclerosis, cancer, and inflammatory bowel disease [21,22,23]. The authors also found that TNF-α, as a proinflammatory cytokine, promotes the expression of miR-155 in human adipocytes via nuclear factor-κB (NF-κB). Further, it has been shown that probiotics exert an anti-inflammatory effect by reducing the expression of miR-155 in mice with colitis and can also decrease the expression of miR155 in colorectal cancer, along with its target gene Kirsten rat sarcoma virus (KRAS) [24,25]. To the best of our knowledge, this is the first time that the effects of probiotics on miR-155 expression in obese patients have been shown. As we showed, 12 weeks of supplementation with the new probiotic approach resulted in significantly lower expression of miR-155-5p compared to the control group, indicating an anti-inflammatory potential of this probiotic combination. Interestingly, recent studies have shown that serum miRNA-155 is significantly overexpressed in women with polycystic ovary syndrome (PCOS) [26,27], an endocrine disease which affects 6% to 20% of women of reproductive age, with a high prevalence of obesity/overweight in women with PCOS. Cirillo et al. found that miR-155 expression was significantly upregulated in ovarian granulosa cells, while miR-155 showed a trend to lower expression in follicular fluids from PCOS patients compared to controls [28]. Considering that probiotic supplementation has been shown to significantly improve metabolic parameters, hormonal profile, and inflammatory indicators in patients with PCOS [29], as well as results from our study, probiotics could be suggested as a new treatment approach for PCOS. Moreover, our study showed a significant correlation between the expression of miR-155-5p and BMI, which additionally supports the important role of miR-155 in the treatment of obesity and obesity-related diseases.

The repressing role of miR-125b-5p in adipogenesis has been recognized, as its expression was found to be suppressed in obese patients [30]. Furthermore, literature data suggest that miR-125b-5p reduces triglyceride accumulation in adipocytes, and its overexpression in preadipocytes plays a protective role in oxidative stress [31]. Interestingly, Ortega et al. have shown that surgically induced weight loss can affect plasma miRNA levels in morbidly obese patients, leading to a significant decrease in miR-125b [32]. In our study, we observed a significant decrease in miR-125b-5p levels in the intervention group after 12 weeks. Although miR-125b-5p expression decreased in the intervention group, no statistical differences were observed compared to the placebo group (Table 4). To the best of our knowledge, no study has investigated the role of probiotics in regulating miR-125b-5p, and this result deserves further investigation.

According to the literature data, miRNA-126-3p has an anti-inflammatory effect [33], and it plays a very important role in endothelial function, leading to enhanced endothelial proliferation and atheroprotection [34]. Wang et al. investigated the effect of exercise and dietary intervention in obese adults on the expression of miRNAs pivotal for endothelial function, as obesity is often associated with increased cardiovascular morbidity, including endothelial destruction [7]. The results showed that circulating levels of miR-126 were significantly upregulated by exercise and a dietary intervention, leading to an improvement in endothelial function. Our study showed a decreasing trend of miR-126-3p in the intervention group after 12 weeks of supplementation, although it was not statistically significant. An upregulation was observed within the placebo group (Table 4). This could be due to the fact that the control group did not receive probiotics and that the low-grade inflammation specific to obesity may have been higher than in the intervention group. Considering the data indicating that miR-126 is overexpressed in inflammatory bowel disease (IBD), where the gut microbiota is disturbed, the probiotic intervention probably improved the gut microbiota balance and prevented changes in the expression of miR-126-3p. 

Regarding miR-34a, no significant differences were observed between groups or time points after the 12-week intervention. A study by Fu et al. conducted on obese individuals suggested a role of miR-34a in regulating white adipose tissue browning [35]. Therefore, further studies are needed to clarify the effect of probiotics on miR-34a expression, particularly on the possible association between probiotics’ effect on browning, as a well as a potential approach in obese individuals.

The miR-26 family (miR-26a-1, miR-26a-2 and miR-26b) represents a very important miRNA family involved in many developmental and physiological functions, such as neuronal differentiation, muscle development, and hepatic glucose and lipid metabolism [36,37,38]. A recent in vivo study discovered that loss of the miR-26 family lead to the expansion of adipose tissue, demonstrating its role in adipogenesis. Specifically, it was shown that decreased expression of miR-26b in visceral adipose tissue (VAT) may be involved in obesity-induced insulin resistance [39,40]. The notion that miR-26b may be an important mediator in regulating obesity-related insulin sensitivity and inflammatory responses was additionally confirmed by Xu et al., who found that TNF-α, leptin, and resistin treatment downregulated the expression of miR-26b in adipocytes [41]. However, we have not detected any significant effect of probiotic intervention on miR-26b-5p expression.

Of particular interest was also to examine the effect of the new probiotic concept on the expression of miRNA-24, which is highly expressed in hepatocytes and plays an important role in adipose tissue functions such as lipolysis, glucose and glycerol turnover, and insulin sensitivity [42,43]. Insulin-induced gene 1 (*Insig1*), an inhibitor of lipogenesis, a process which is a crucial part of adipogenesis, appears to be an important target for miR-24 action. In addition, levels of miR-24 in abdominal adipose tissue were found to be elevated in patients with obesity and T2D [43]. Moreover, miR-24-3p is highly expressed in obese children, thus having an important role in the onset and development of childhood obesity [44]. According to our results, 12-week supplementation with the new probiotic concept resulted in significantly lower expression of miR-24-3p compared to the control group; therefore, this combination may have a beneficial effect in the treatment of obesity and diabetes. Considering the negligible side effects of probiotics, these findings deserve special attention and additional research with the aim of applying the new concept as a complementary treatment in obese children. In addition, we observed a correlation between miR-24-3p and TC levels. More precisely, patients who, after the intervention, had lower miR-24-3p expression also had lower TC. These results are promising, since it is showed that miR-24 levels are elevated in patients with familial hypercholesterolemia and obesity-associated hyperlipidaemia in HFD-fed mice [45].

SIRT1 is able to mediate beneficial effects of probiotic in obese models. Several mechanisms are proposed, showing that SIRT1 inhibits adipogenesis by suppressing peroxisome proliferator-activated receptor-γ (PPAR-γ), the major regulator of adipogenesis [46]. Also, Khalili et al. showed that two months of supplementation with *Lactobacillus casei* resulted in significant weight loss and markedly increased *SIRT1* expression in patients with T2D and reduces appetite and body weight [10]. In our study, we did not find significant effects of probiotic intervention on expression compared to the control group, although *SIRT1* expression was decreased in the control group (*p* = 0.035). These results could be explained by the fact that the regulated gut microbiota in the intervention group did not allow for a reduction in *SIRT1* expression that occurred in the placebo group, which is supported by the literature finding that gut tissues from patients with a disturbed gut microbiota, such as in ulcerative colitis, expressed significantly lower levels of *SIRT1* than healthy controls [47].

In the early 1990s, Hotamisligil et al. demonstrated that the adipose tissue of obese patients had elevated levels of *TNF-α* and the corresponding protein [48]. Several additional studies confirmed that inflammation is critically involved in the pathogenesis of insulin resistance and obesity. Therefore, TNF-α has been referred to as the main mediator between obesity-induced inflammation and insulin resistance [49]. In our study, we observed a decrease in *TNF-α* in the intervention group after the probiotic supplementation. Although a slight decrease in *TNF-α* level was observed compared to the placebo group, these differences were not statistically significant. Our results suggest that administration of the probiotic concept could reduce TNF-α secretion/expression and improve inflammatory status in obese individuals by modulating the gut microbiota. Therefore, TNF-α activation pathways might be the primary target of the anti-inflammatory effect of probiotics.

The effect of the intervention was also investigated on the expression of pyruvate dehydrogenase kinase 4 (*PDK4*), which has been identified as the main regulator of the activity of the pyruvate dehydrogenase complex (PDC), an important controller of glucose oxidation [50]. In our study, we found no significant effect of the 12-week intervention on *PDK4* expression. A possible explanation for the unaffected levels of *PDK4* expression could be the lack of significant weight loss during the 12-week intervention. To date, there is no study investigating the role of probiotics in regulating *PDK4* in obese women.

Although the results of the study appear to be innovative, this study has limitations, particularly the relatively small sample size, which restricts the generalization of the results and may explain the lack of statistical significance for most parameters. Nevertheless, further research in this direction and with a larger number of participants of both sexes is essential for drawing more precise conclusions.

## 5. Conclusions

To the best of our knowledge, this is the first pilot study reporting the effects of a specific probiotic product on epigenetic markers in overweight and obese women. The results of our pilot study show that, although the intervention with the novel probiotic product did not reduce body weight or regulate lipid status in the obese female participants, a positive effect was observed in regulating the expression of certain miRNAs (e.g., miR-155-5p) and mRNAs (e.g., *TNFα*) important in the regulation of inflammation and adipogenesis. This suggests a potential mechanism for long-term health effects. 

## Figures and Tables

**Table 1:** Dietary habits of participants.

Group	Processed Foods	Fruits and Vegetables	Meat	Fish	Dairy Products	Grain Products	Sweets	Physical Activity	Exercise
Intervention group (N = 12)	Rarely (58.33%)	Every day (75%)	1–6 p/w (75%)	Rarely (41.67%) 1 p/w (41.67%)	Occasionally (54.55%)	Rarely (60%)	1–3 p/w (66.67%)	2–5/w (58.33%)	Rarely (66.67%)
Placebo group (N = 8)	Rarely (50%)	Every day (87.5%)	1–6 p/w(50%)	Rarely (37.5%) 1 p/w (37.5%)	Every day (66.67%)	Rarely (50%)	1–3 p/w (50%)	6–7/w (62.5%)	Rarely (87.5%)

p/w = Portions per week; number/week = times per week.

**Table 2:** Relevant anthropometric characteristics of participants.

	T0		T1		Level of Changes within the Group † Δ (%)	
Parameter	Intervention Group (N = 12)	Placebo Group (N = 8)	Intervention Group (N = 12)	Placebo Group (N = 8)	Intervention Group (N = 12)	Placebo Group (N = 8)
Weight (kg)	97.76 ± 13.47	104.86 ± 15.94	95.20 ± 14.46	101.88 ± 14.11	−2.67 ± 5.07	−2.64 ± 4.28
	*p* = 0.297, Part. Eta^2^ = 0.06		*p* = 0.321, Part. Eta^2^ = 0.055		*p* = 0.987	
BMI (kg/m²)	35.3 ± 5.10	39.6 ± 8.14	34.39 ± 5.72	38.5 ± 7.36	−2.76 ± 4.99	−2.55 ± 4.47
	*p* = 0.162, Part. Eta^2^ = 0.106		*p* = 0.177, Part. Eta^2^ = 0.099		*p* = 0.926	
Body fat mass (%)	42.34 ± 5.44	45.53 ± 4.39	42.06 ± 4.78	45.79 ± 5.51	−0.4 ± 4.85	0.45 ± 5.2
	*p* = 0.198, Part. Eta^2^ = 0.101		*p* = 0.144, Part. Eta^2^ = 0.129		*p* = 0.725	

† Delta value is calculated according to the formula: ((T1/T0) − 1)·100%. Partial Eta^2^ = 0.01 indicates a small effect, Partial Eta^2^ = 0.06 indicates a medium effect, Partial Eta^2^ = 0.14 indicates a large effect. *p* ≤ 0.05, statistical significance.

**Table 3:** Changes in lipid status before and after 12 weeks of intervention.

	T0		T1		Level of Changes within the Group † Δ (%)	
Parameter	Intervention Group (N = 12)	Placebo Group (N = 8)	Intervention Group (N = 12)	Placebo Group (N = 8)	Intervention Group (N = 12)	Placebo Group (N = 8)
TC (mg/dL)	5.27 ± 1.06	4.9 ± 0.83	6.013 ± 1.64	4.70 ± 0.33	4.50 ± 9.79	1.08 ± 12.93
	*p* = 0.440, Part. Eta^2^ = 0.038		*p* = 0.079, Part. Eta^2^ = 0.218		*p* = 0.570	
HDL (mg/dL)	1.23 ± 0.32	1.25 ± 0.32	1.28 ± 0.47	1.35 ± 0.44	4.63 ± 13.19	2.54 ± 10.84
	*p* = 0.889, Part. Eta^2^ = 0.001		*p* = 0.783, Part. Eta^2^ = 0.006		*p* = 0.763	
LDL (mg/dL)	3.54 ± 1.02	2.98 ± 0.75	3.91 ± 0.98	2.87 ± 0.67	4.17 ± 13.06	2.09 ± 20.40
	*p* = 0.237, Part. Eta^2^ = 0.092		*p* = 0.051, Part. Eta^2^ = 0.304		*p* = 0.832	
TG (mg/dL)	1.88 ± 1.15	1.46 ± 0.9	1.50 ± 0.62	1.07 ± 0.24	3.54 ± 61.32	3.34 ± 41.84
	*p* = 0.426, Part. Eta^2^ = 0.043		*p* = 0.131, Part. Eta^2^ = 0.166		*p* = 0.991	

† Delta value is calculated according to the formula: ((T1/T0) − 1)·100%. Partial Eta^2^ = 0.01 indicates a small effect, Partial Eta^2^ = 0.06 indicates a medium effect, Partial Eta^2^ = 0.14 indicates a large effect. *p* ≤ 0.05, statistical significance.

**Table 4:** Changes in miRNA and mRNA values before and after 12 weeks of intervention.

	T0		T1	
Parameter	Intervention Group (N = 12) Δct	Placebo Group (N = 8) Δct	Intervention Group (N = 12) Δct	Placebo Group (N = 8) Δct
miR-34a-5p	11.34 ± 1.20	11.46 ± 1.55	11.97 ± 2.42	11.01 ± 1.32
	*p* = 0.889, Part. Eta^2^ = 0.002		*p* = 0.356, Part. Eta^2^ = 0.057	
miR-155-5p	8.69 ± 1.31	9.15 ± 1.13	9.3 ± 0.85 *	8.38 ± 1.06
	*p* = 0.425, Part. Eta^2^ = 0.036		***p* = 0.050**, Part. Eta^2^ = 0.205	
miR-125b-5p	5.41 ± 1.18	6.43 ± 1.11	5.99 ± 1.36 *	5.85 ± 1.02
	*p* = 0.068, Part. Eta^2^ = 0.173		*p* = 0.805, Part. Eta^2^ = 0.003	
miR-26b-5p	1.51 ± 0.44	2.17 ± 0.63	1.84 ± 0.53	1.92 ± 0.35
	***p* = 0.013**, Part. Eta^2^ = 0.295		*p* = 0.720, Part. Eta^2^ = 0.007	
miR-126-3p	1.88 ± 0.56	2.42 ± 0.60	2.26 ± 0.65	2.13 ± 0.55 *
	*p* = 0.059, Part. Eta^2^ = 0.184		*p* = 0.651, Part. Eta^2^ = 0.012	
miR-24-3p	3.13 ± 0.76	3.63 ± 1.1	3.42 ± 0.38	2.71 ± 0.97
	*p* = 0.247, Part. Eta^2^ = 0.074		***p* = 0.031**, Part. Eta^2^ = 0.233	
*PDK4*	11.2 ± 0.74	11.18 ± 0.47	11.00 ± 0.67	10.97 ± 0.58
	*p* = 0.957, Part. Eta^2^ = 0.00		*p* = 0.916, Part. Eta^2^ = 0.001	
*SIRT1*	10.10 ± 0.87	10.12 ± 0.48	10.52 ± 0.63	10.38 ± 0.52 *
	*p* = 0.915, Part. Eta^2^ = 0.001		*p* = 0.635, Part. Eta^2^ = 0.014	
*TNF-α*	10.24 ± 1.66	10.46 ± 0.94	11.36 ± 1.12 *	10.89 ± 0.82
	*p* = 0.754, Part. Eta^2^ = 0.007		*p* = 0.341, Part. Eta^2^ = 0.057	

* *p* ≤ 0.05, statistical significance within the group.

## Data Availability

Data is unavailable due to ethical restrictions.
